# Medication Management after Bariatric Surgery: Providing Optimal Patient Care

**DOI:** 10.3390/jcm9051511

**Published:** 2020-05-17

**Authors:** Daniel Porat, Arik Dahan

**Affiliations:** Department of Clinical Pharmacology, School of Pharmacy, Faculty of Health Sciences, Ben-Gurion University of the Negev, Beer-Sheva 84105, Israel; poratdan@post.bgu.ac.il

**Keywords:** bariatric surgery, bioavailability, biopharmaceutics, drug absorption, drug solubility, intestinal permeability

## Abstract

Substantially altered gastrointestinal anatomy/physiology after bariatric surgery presents new challenges for the proper medication management of these patients; drug absorption and bioavailability may increase, decrease, or remain unchanged post surgery, depending on the specific drug in question and the type of bariatric procedure. In this article, we offer a concise overview of the various aspects of this clinically significant issue, aiming to provide readers with a clear understanding as well as practical tools to handle drug management post bariatric surgery. Realizing the potentially altered pharmacokinetics of various drugs after bariatric surgery is essential for providing optimal pharmacological therapy and overall patient care.

## 1. Introduction

The global rise of the obesity epidemic is amongst the toughest challenges we face. The many comorbidities associated with severe obesity (type 2 diabetes, hyperlipidemia, hypertension, heart disease, stroke, cancer, depression, and many others) have turned this disease into the second most common factor contributing to preventable death (second only to tobacco) [1]. The price tag for treating obesity and related conditions is daunting. Diet/exercise strategies alone are difficult to maintain in the long-term, and at present, pharmacological treatment for obesity is associated with only modest weight loss and various adverse effects. To date, bariatric surgery, which aims to limit caloric intake, decrease nutrient absorption, or both, is the most effective solution for severe obesity with comorbidities, and the number of patients undergoing bariatric surgery is rapidly and constantly growing worldwide [2].

**Figure 1:**
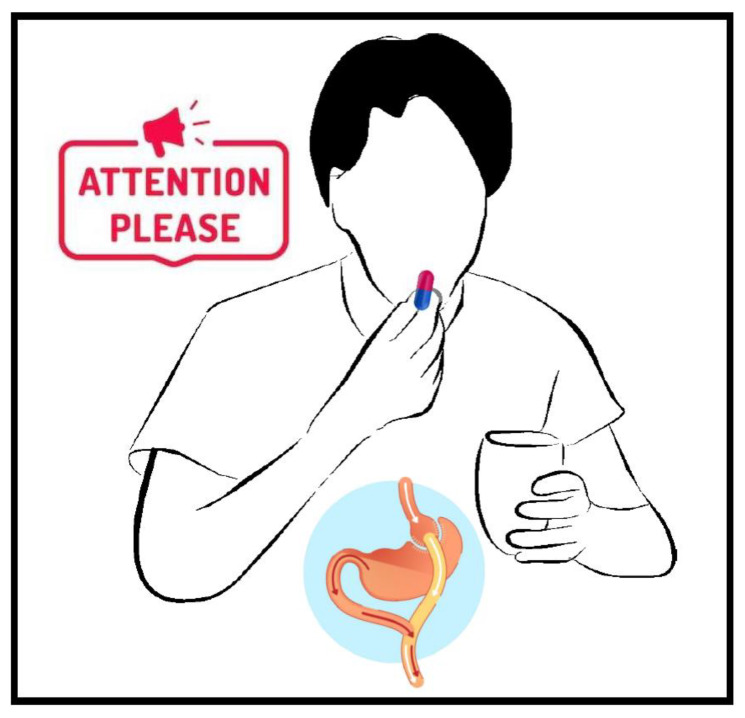
Illustration of the complexity behind drug management of patients after bariatric surgery; accounting for substantially altered gastrointestinal anatomy and consequent potential altered drug absorption/bioavailability is essential for providing optimal pharmacological therapy and overall patient care.

This rapidly growing population of bariatric patients presents new challenges to the field of oral drug therapy (Figure 1). Substantially altered gastrointestinal (GI) anatomy may greatly influence the absorption of drugs following oral administration, with potentially significant clinical implications [3]. The complex process of drug absorption involves multiple stages, and many of them may be affected by bariatric surgery, due to physiological factors, drug-related physicochemical factors, and factors associated with the dosage form (e.g., solid vs. liquid drug product). Overall, drug absorption and bioavailability may increase, decrease, or remain unchanged post surgery, depending on the specific drug in question and the type of bariatric procedure [4]. With years of morbid obesity and comorbidities, many bariatric patients are likely to consume multiple drugs for various medical conditions. Many drugs, from different pharmacological classes, were reported to be influenced by bariatric procedures, including some vital and essential drugs, e.g., antiepileptic agents [5], immunosuppressants [6], tyrosine kinase inhibitors [7,8], antiretroviral therapy [9], psychiatric medications [10,11], hormone replacement therapy [12], pain medications [13,14], and others. Realizing the potentially altered pharmacokinetics of various drugs after bariatric surgery is hence essential for providing optimal pharmacological therapy and patient care.

To date, the most commonly performed bariatric procedures are the sleeve gastrectomy, the single-anastomosis gastric bypass, and the Roux-en-Y gastric bypass (RYGB) [15]. While the first involves only the stomach and limits food intake, the latter two involve the stomach and the small intestine, and limit both food intake and nutrient absorption. The exact type of bariatric procedure directly influences the potential for altered oral drugs pharmacokinetics, and hence this is a major factor when analyzing the drugs taken by a specific bariatric patient.

## 2. Potential Mechanisms

Pharmacokinetic alterations of oral drugs after bariatric surgeries may occur in many different mechanisms. After being swallowed, solid immediate-release drug products have to disintegrate and be broken down into small particles. This process typically happens in the stomach, and since all bariatric procedures involve significantly reduced stomach size and contractility, tablets may fail to adequately disintegrate after bariatric surgery. Similarly, drug dissolution in the GI is a prerequisite for subsequent absorption, and for many drugs with borderline solubility, the reduced stomach size (and hence fluid intake) and contractility may result inadequate drug dissolution. Moreover, the solubility/dissolution of many drugs is pH-dependent and the increased gastric pH after bariatric surgeries (attributable to decrease in acid-producing parietal cells) may further alter their dissolution. After gastric emptying into the duodenum, lipophilic drugs may require bile and pancreatic secretions for solubility/dissolution [16], while in some malabsorptive bariatric procedures (e.g., RYGB) upper small intestinal segments are bypassed, and these secretions are diverted to lower segments, which may hamper drug solubilization. In the next step towards absorption, solubilized drug molecules have to permeate across the gut membrane into the enterocytes [17]. Many drugs require the entire small intestinal length, surface area, and transit time, to achieve adequate absorption, and since bypass procedures reduce all three parameters, hampered absorption may result. Furthermore, this permeation may be a passive process based on simple diffusion across the enterocyte [18], or active carrier-mediated process, that may work in both uptake/efflux directions; the expression of these transporters may be region-dependent, and hence, malabsorptive bariatric procedures that bypass significant portion of the small intestine may change the exposure of drugs to relevant transporters, thereby changing their absorption profile [19]. Likewise, the expression of metabolic enzymes along the gastrointestinal tract may be region-dependent, and bypassing the upper intestine by malabsorptive procedures may change the fraction of dose that escapes pre-systemic intestinal metabolism; CYP450-3A4 is such enzyme, with its highest expression level in the upper small intestine, and since this is the major intestinal drug metabolizing enzyme, the bioavailability of relevant drugs may increase after bypass surgeries [20]. Immediate alterations in gut microbiota following bariatric procedures were also reported to potentially impact drug exposure [21]. Next, drug molecules pass through the liver before reaching the systemic circulation and may undergo presystemic hepatic metabolism. This process may also be affected by bariatric surgery; the reduced liver size attributable to the rapid weight loss may cause decreased hepatic metabolism and increased bioavailability. Significant loss of adipose tissue post surgery may then change drug’s distribution and pharmacokinetics [22,23]. Finally, renal function is altered in patients with obesity, and after substantial weight changes; the limited fluid intake after bariatric surgery can further impair renal function, with potentially reduced excretion and increased overall exposure of relevant drugs. It should be noted that GI adaptation processes take place over time [24], making the first 1–2 years post surgery a timeframe more prone to PK changes.

## 3. Clinical Recommendations

General recommendations for oral drug therapy after bariatric surgery have been outlined in guidelines [25,26]. First and foremost, plasma drug levels, clinical outcomes, and laboratory markers should be frequently monitored, especially with drugs that require periodic plasma level control. Oftentimes, oral liquid drug products may be available to replace solid dosage forms, and this is advisable for the first 2 months post surgery; it should be ascertained that the liquid product does not contain nonabsorbable sugars due to dumping syndrome risk. In case no liquid product is available on the market for a certain drug, patients may be advised to crush (tablet) or open (capsule) the solid dosage form during these first two months; it should be noted that this is irrelevant for controlled-release products. Alternatively, non-oral dosage forms may be used to avoid complications, especially for drugs with critical effects on patient’s condition. Special attention should be paid to drugs that need food or acidic environment for their absorption (e.g., carbamazepine, phenytoin). Drug-class-specific recommendations include extra care in diabetes therapy, including avoiding drugs with hypoglycemia risk (e.g., sulfonylurea, meglitinides), and adjusted dose of insulin and other antidiabetics until normal glucose levels are achieved and maintained. Diuretics should be used cautiously, since together with the surgery’s diuretic effect there is increased risk of dehydration. NSAIDs and corticosteroids should be avoided due to risk of gastric damage. Oral contraceptives should be switched to non-oral options due to reduced efficacy. Overall, it is necessary to carefully instruct the patients on their revised drug regimen; contribution of a trained clinical pharmacist at this critical point is prudent.

## 4. Conclusions

Altogether, the complexity described in this article, along with the substantial inter- and intrapatient variability in drug absorption post surgery, and the limited data available on this subject emphasize the urgent need for further research in order to reveal the optimal oral drug therapy after bariatric surgery. All medical staff must be aware of this critical concern, including physicians, pharmacists, nurses, and dieticians, so the short- and long-term safety and efficacy of bariatric patients’ drug regimen can be ensured.
